# Progress in Understanding Mitochondrial Disorders

**DOI:** 10.4103/0974-9233.51991

**Published:** 2008

**Authors:** Anuradha Ganesh, Govindaswamy Kumaramanickavel

**Affiliations:** 1 Department of Ophthalmology, Sultan Qaboos University Hospital, Muscat, Oman; 2 SNONGC Department of Genetics and Molecular Biology, Vision Research Foundation, Sankara Nethralaya, Chennai, India. Fax: +91-44-28254180; Email: gkumarvel@gmail.com

In this edition of *MEJO*, Al-Enezi et al, review mitochondrial disorders with significant ophthalmic manifestations.^1^ Their effort is timely due to major recent breakthroughs in the understanding of mitochondrial diseases, with the discovery of an impressive and ever-increasing number of mutations of mitochondrial DNA (mtDNA) providing new pathogenic insights in this rapidly expanding area of human pathology.

The term “mitochondrial disorder” describes defects in the mitochondrial electron transport chain. The first disorder of mitochondrial function was described by Luft in 1959.^2^ Over the ensuing decades, multiple cases of mitochondrial dysfunction have been reported.

Mitochondria contain their own DNA – the mtDNA; the complete sequence of which was reported in 1981.^3^ Mitochondrial disorders are caused by mutations in mtDNA or nuclear genes, or both, that regulate the formation and maintenance of an intact oxidative phosphorylation system in the mitochondria.^4^ Since human mtDNA is strictly maternally inherited, in familial cases of mitochondrial diseases, inheritance is nonmendelian and passes from mother to offspring. Mothers with a higher concentration of mutated mtDNA are more likely to have clinically affected children. Families with a mendelian pattern of inheritance (autosomal dominant) of mitochondrial gene defects appear to be attributable to defects in the nuclear genes that encode proteins responsible for mtDNA replication or the maintenance of mitochondrial genomes. Currently, 200 pathogenic point mutations, deletions, insertions, and rearrangements of the mitochondrial genome are known.^5^ Individuals with mtDNA disease often harbour a mixture of mutated and wild (normal) mtDNA (heteroplasmy). The disease is manifested only when a certain threshold of abnormal mitochondria is crossed.

Mitochondrial diseases can present at any age. These disorders present with a bewildering array of clinical presentations. Post mitotic tissues as neurons, cardiac muscles, skeletal muscles, liver, kidney and endocrine organs have high energy demands, are therefore highly sensitive to effects of mutated mtDNA, and are often clinically involved. The neuromuscular findings include involvement of the central nervous system (altered level of consciousness, developmental delay, seizures, involuntary movement), the peripheral nervous system (peripheral neuropathy and decreased deep tendon reflexes), and muscular system (hypotonia, muscle weakness, and muscle pain).^6^ Most patients with neuromuscular symptoms will have either normal or only slightly elevated (2X normal) serum creatine kinase. The results of electromyography and nerve conduction studies are also usually normal. The unexpected finding of a normal creatine kinase level or normal electromyography in a patient with significant muscle weakness is a clue to search for a mitochondrial disorder. Approximately 20 percent of patients demonstrate intellectual dysfunction or psychiatric disturbances. Ten percent demonstrate hepatic signs. Cardiac presentations include arrhythmia, cardiomyopathy, cardiac murmur, or sudden death.^6^,^7^

Numerous studies have associated mitochondrial diseases with ophthalmic manifestations that include progressive external ophthalmoplegia (PEO) with ptosis, bilateral optic neuropathy, pigmentary retinopathy, and retrochiasmal visual loss.

Corneal clouding and cataracts are infrequent ophthalmic manifestations of mitochondrial disease. The retinal pigment epithelium is highly vulnerable to be involved by mtDNA defects. The retinopathy is phenotypically variable and frequently subclinical and depends to some extent on the specific type or site of mtDNA mutation.^8^

In the majority of situations, the diagnosis of a mitochondrial disorder remains a clinical diagnosis. The suspicion of a mitochondrial disorder should be based not only on an awareness of the presenting signs, but on having a high index of suspicion in any patient presenting with multisystem involvement. Over the last few decades, several sets of diagnostic criteria based on some combination of clinical, laboratory, pathologic, and genetic findings have been developed to assist in the recognition and diagnosis of mitochondrial disorders.^9^ The most recognized laboratory abnormality in patients with mitochondrial disorders is lactic acidosis. Lactate may be elevated in cerebrospinal fluid, even in the presence of normal venous values. A muscle biopsy in patients with suspected mitochondrial disorders characteristically shows ragged-red fibers (RRFs). This provides an important clue to the diagnosis. RRFs are characterized by large proliferations of subsarcolemmal mitochondria, which appear red due to Gomori-Trichrome staining. Since mitochondrial disorders are progressive, muscle pathology is likely to be normal in early stages of the disease. Repeated sampling of muscle tissue may therefore be necessary to detect abnormalities. RRFs are not pathognomonic of mitochondrial diseases, but can also be seen in other conditions like dystrophies, dermatomyositis and in older individuals. However, in these diseases, the intensity and the proportion of muscle biopsies showing RRFs will be sparse. Presence of more than 2 percent RRFs in skeletal muscle biopsy is taken as one of the criteria for the diagnosis of mitochondrial disease.^9^ Electron microscopy typically reveals abundant mitochondria with abnormal appearance with disorganized cristae. Neuroradiologic features are nonspecific and include symmetric basal ganglia calcifications, brain infarcts, or cerebral and cerebellar atrophy.^10^ Finally, Southern blotting may reveal the common deletion. Direct sequencing of mitochondrial genome from a muscle biopsy specimen can be helpful.

Although there has been an explosion of studies on the mitochondrial diseases over the past few years, therapy remains very limited. Genetic counseling may be appropriate for certain diseases. Appropriate clinical interventions to prevent the known complications of mitochondrial diseases include cardiac pacing, surgical correction of ptosis, and cataract surgery. Antioxidants may be beneficial to patients as free radicals are proposed to be important in the pathogenesis of mitochondrial disorder.
